# Open technique for supra-acetabular pin placement in pelvic external fixation: a cadaveric study

**DOI:** 10.1186/s10195-022-00635-w

**Published:** 2022-03-14

**Authors:** Sukanis Chumchuen, Wissarut Lertpullpol, Adinun Apivatgaroon

**Affiliations:** Department of Orthopaedics, Faculty of Medicine, Thammasat University, 99 Moo 18, Khlong Nueng, Khlong Luang, 12120 Pathumthani Thailand

**Keywords:** Open technique, Supra-acetabular pin, Pelvic external fixation, Human cadaver

## Abstract

**Background:**

Standard supra-acetabular pin placement still needs fluoroscopic guidance, which is technically demanding for an untrained surgeon due to the unfamiliar fluoroscopic view and the risk of damaging some structures. The risks associated with the open technique without fluoroscopy have not yet been investigated, despite the palpable entry point and large bony corridor for rapid insertion in the limited time available for the management of unstable pelvic fracture patients. The aim of this study was to compare the open technique without fluoroscopy to the fluoroscopically assisted percutaneous technique for the positioning of supra-acetabular screws in cadavers without pelvic instability.

**Materials and methods:**

The open technique for half-pin placement was compared to standard fluoroscopic guidance in 16 hemipelves (8 cadavers). The open technique was first performed on one side in each cadaver after simple randomization, followed by standard fluoroscopic guidance on the other side. In the open technique group, a Schanz pin 5 mm in diameter and 200 mm in length was inserted in the area just above the anteroinferior iliac spine (AIIS) and aimed with a medial inclination of 20° and a cephalad inclination of 10–20° after a 2 cm pilot drill hole had been established. Standard fluoroscopically guided pin placement was performed on the other side. Fluoroscopic assessment was conducted after final pin placement on both sides. The lateral femoral cutaneous nerve of the thigh (LFCN) and the hip capsule were identified via the Smith–Peterson approach. After complete dissection of soft tissue, it was clearly apparent that pin penetration was conducted outside the bony corridor.

**Results:**

The LFCN was found to be in a risk zone near the pin (mean distance, 15 mm; range, 0–30 mm). One LFCN may have been injured in the fluoroscopic guidance group. The mean medial inclination of the pin was 19.8° (range, 5–40°) and the mean cephalad inclination was 11.5° (range 0–20°) in the open technique group. The mean medial inclination of the pin was 30.4° (range, 20–45°) and the mean cephalad inclination was 19.3° (range, 2–35°) in the fluoroscopic guidance group. The mean distance of the pin entry point from the AIIS was 11.1 mm (range, 0–35 mm) in the open technique group. The mean distance of the entry point of the pin from the AIIS was 15.1 mm (range, 0–25 mm) in the fluoroscopic guidance group. The mean hip joint capsule distance was 12 mm (range, 8–25 mm). No joint penetration was observed in the open technique group, compared to one joint penetration in the fluoroscopic guidance group. No sciatic notch penetration was found in either group, but pin penetration outside the external cortex of the ilium was found only in the open technique group, in 4 hemipelves.

**Conclusions:**

This study shows that the freehand technique performed by experienced trauma surgeons may be as acceptable as controlled pin insertion under image intensification for selecting the proper entry point and stabilizing the anterior pelvic ring.

## Introduction

Pelvic external fixation is a life-saving procedure in patients with unstable pelvic injuries, and needs to be applied quickly and safely to reduce the mortality rate in severely multiply injured patients who need stabilization of a pelvic ring fracture. The use of a pelvic binder or external fixator is a useful step in the temporary stabilization of an unstable pelvis with unstable hemodynamics before proceeding to definitive management [1]. Recently, the pelvic binder has rapidly gained in popularity, and it has the advantages of immediate application at the scene and ease of application, which make it superior to an external fixator in the early resuscitation phase and the prehospital setting [2]. However, nursing care in the hospital is difficult in these patients, and moving patients with an unstable pelvic injury is painful and difficult. A pelvic binder increases the risk of skin sores from compression when applied for a prolonged period [3]. The current recommendation is to remove a pelvic binder as soon as physiologically justifiable, and to consider replacing a binder with external pelvic fixation or definitive pelvic stabilization [1, 3].

Two pin placement locations in pelvic external fixation—the iliac crest and the supra-acetabular region—are widely used to stabilize an unstable pelvic ring. The supra-acetabular pin placement technique is biomechanically superior to an iliac crest pin in controlling the posterior pelvic ring [4], and there is less interference with subsequent laparotomy procedures. However, one limitation when utilizing a supra-acetabular pin is the need for fluoroscopic guidance [5], which is technically demanding for an untrained surgeon because of the unfamiliar fluoroscopic view [6, 7] and the potential risk of injury to structures such as the hip joint [8] (which may lead to septic arthritis [9]), the superior gluteal neurovascular bundle, and the lumbosacral trunk (if the half-pin is advanced towards the sciatic notch inferior to the supra-acetabular bone corridor [10]), as well as unavoidable subcutaneous injury to the lateral femoral cutaneous nerve of the thigh (LFCN) [7, 11]. Compared to iliac crest pin placement, which is more popular due to its palpable entry point without the need for fluoroscopic guidance, there is difficulty in obtaining good bony purchase and in following its small, hourglass-shaped bony corridor pathway [12], making it problematic for achieving adequate stability. Therefore, the use of a supra-acetabular pin is considered to be a better way to get adequate stability due to its larger bony corridor and dense bone, but the safety of the open technique has not yet been reported in published literature. The aim of this study was to compare the open technique without fluoroscopy with the fluoroscopically assisted percutaneous technique for the positioning of supra-acetabular screws in cadavers without pelvic instability.

## Materials and methods

**Fig. 1:**
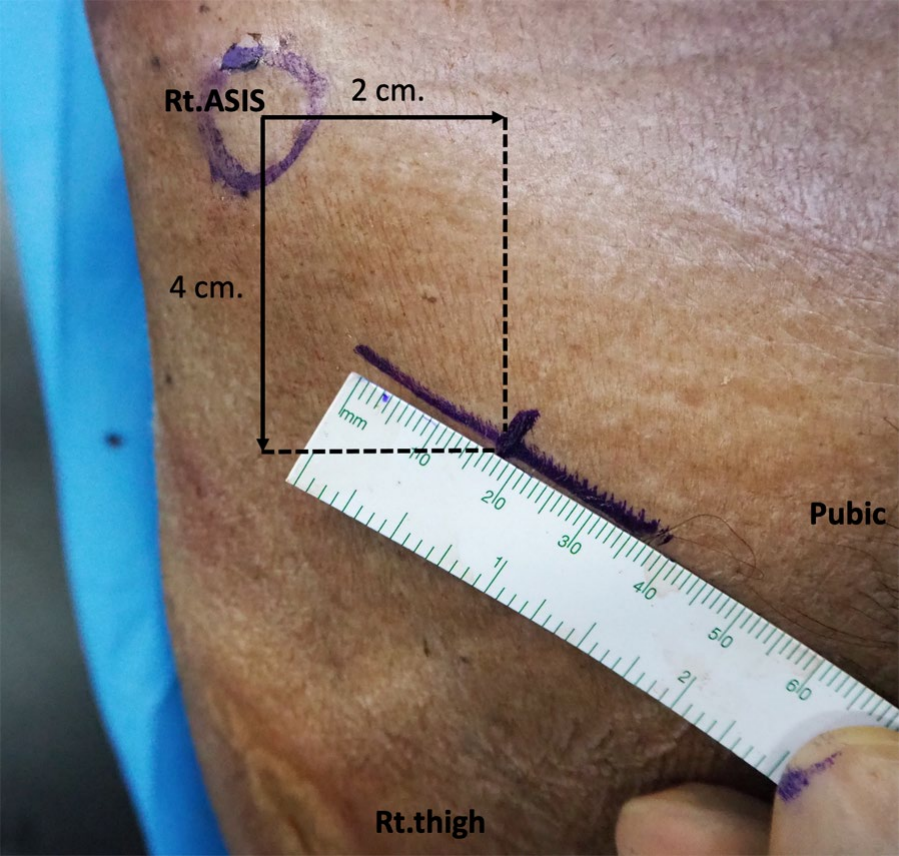
Oblique bikini skin incision centered approximately 4 cm distal and 2 cm medial to the anterior superior iliac spine (or two fingerbreadths distal and one medial to the ASIS) on the right hemipelvis

**Fig. 2:**
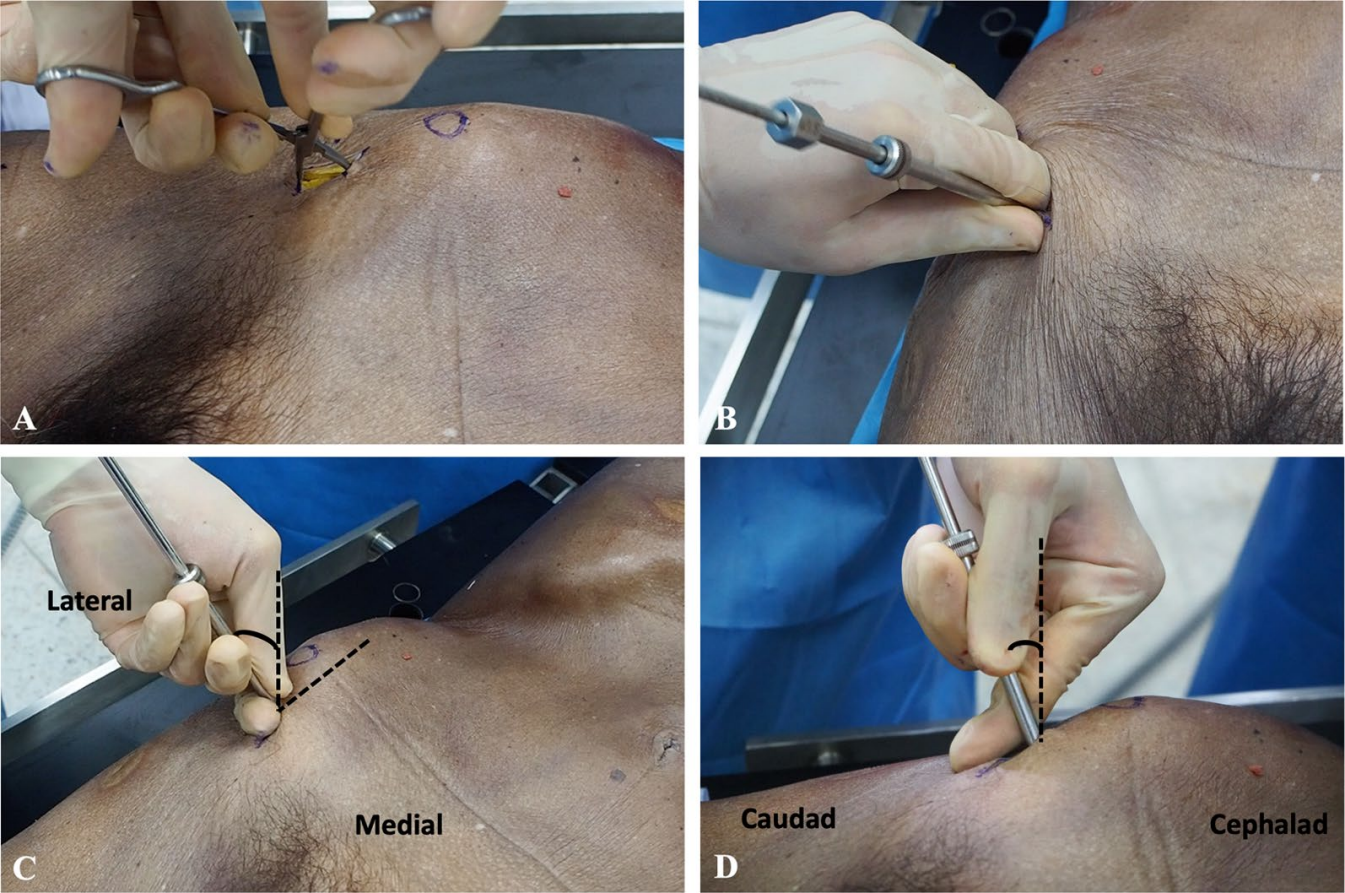
On the right hemipelvis, a longitudinal split of the fascia over the tensor fasciae latae was achieved with artery forceps (**A**), followed by digital blunt dissection until the anterior inferior iliac spine could be palpated, and then a 2 cm pilot drill hole was made using a three-part drill sleeve (**B**). A Schanz screw was inserted at the area just above the AIIS and aimed with a medial inclination of 20° (**C**) and a cephalad inclination of 10–20° (**D**)

**Fig. 3:**
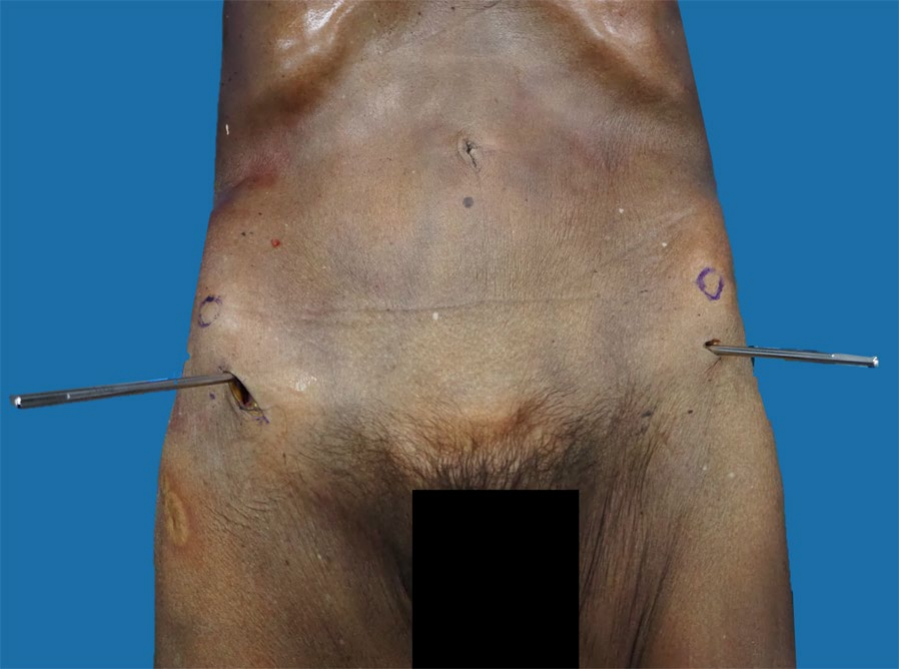
Open-technique pin placement on the right hemipelvis and percutaneous technique on the left hemipelvis

**Fig. 4:**
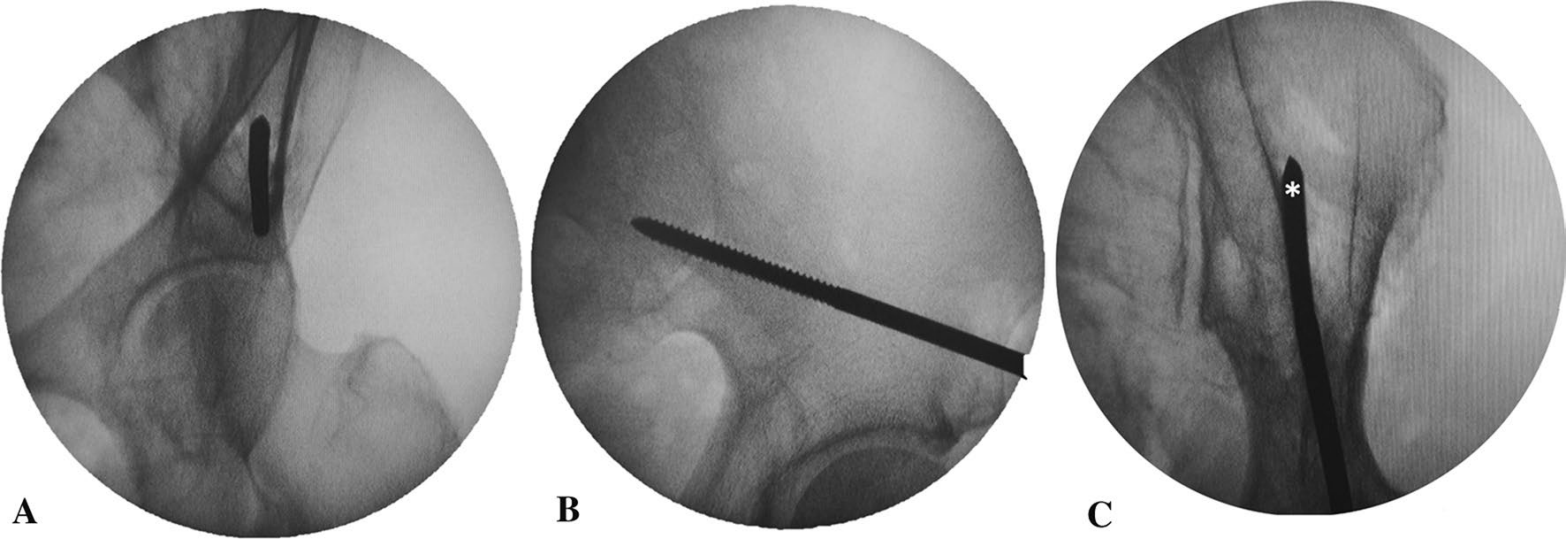
Fluoroscopic image of pin placement on the left hemipelvis in the obturator outlet view (**A**), iliac oblique view (**B**), and obturator inlet view (**C**). The pin entry point is first identified in the obturator outlet view (**A**) in the center of the corridor of bone, and the pin is advanced using the iliac oblique view (**B**), with adjustment of the cephalad inclination angle above the sciatic notch (**A**, **B**: percutaneous technique). Pin penetration outside the lateral cortex (*asterisk*) of the iliac bone was demonstrated on the obturator inlet view (**C**: open technique)

**Fig. 5:**
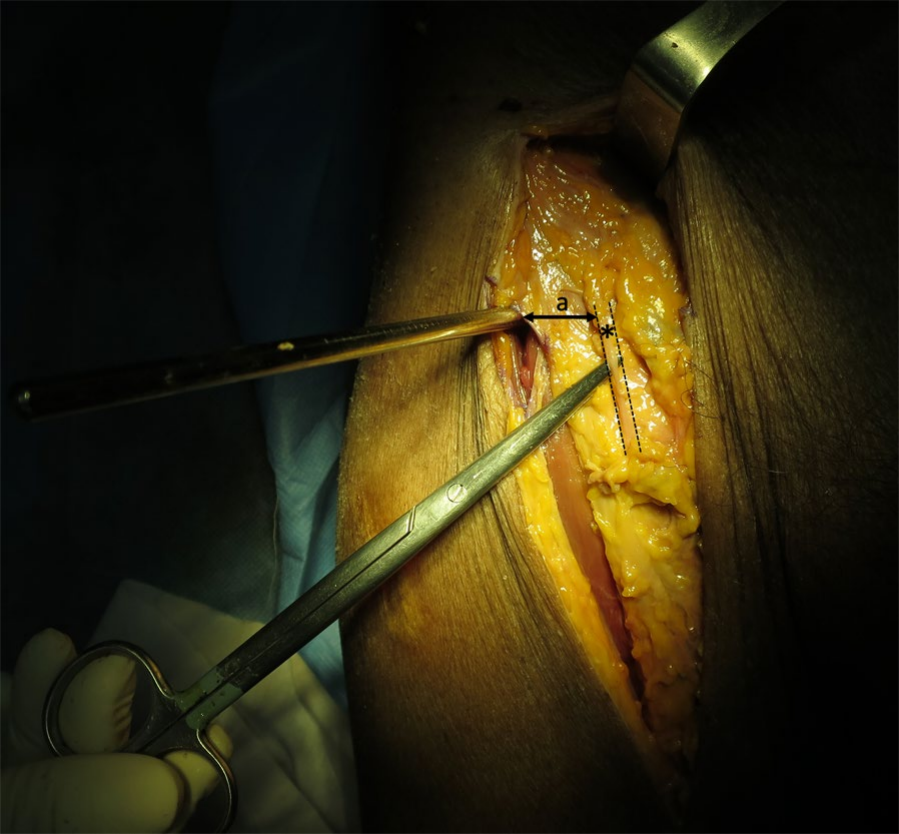
The lateral femoral cutaneous nerve (LFCN) is identified (*asterisk*) medial to the pin on the right hemipelvis, and the distance (*a*) from the pin to the lateral femoral cutaneous nerve (LFCN)

**Fig. 6:**
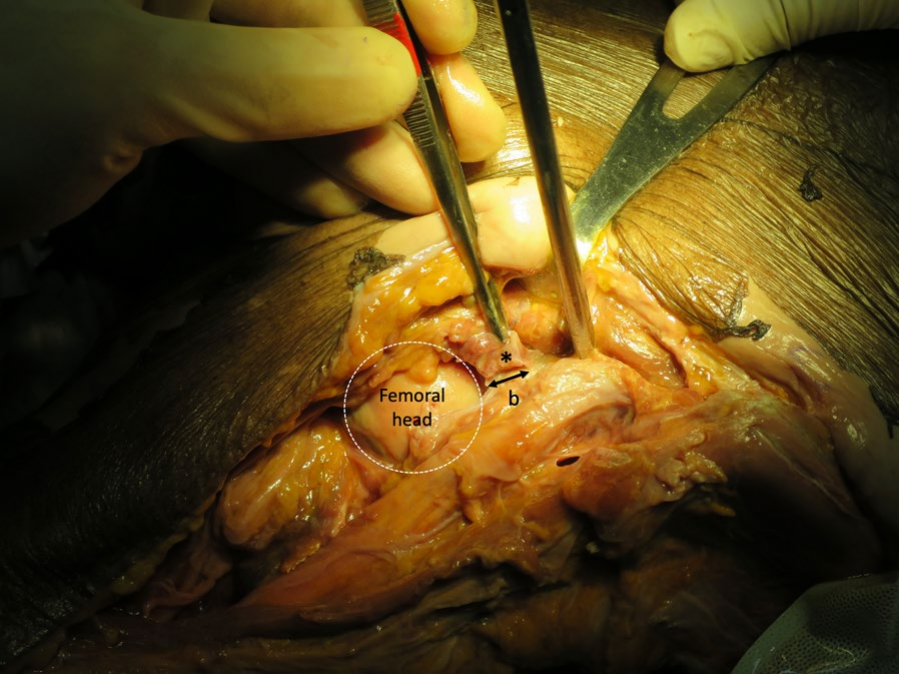
The hip capsule is identified (*asterisk*) distal to the pin insertion site on the left hemipelvis, and the distance (*b*) from the acetabulum edge to the insertion of the hip capsule

**Fig. 7:**
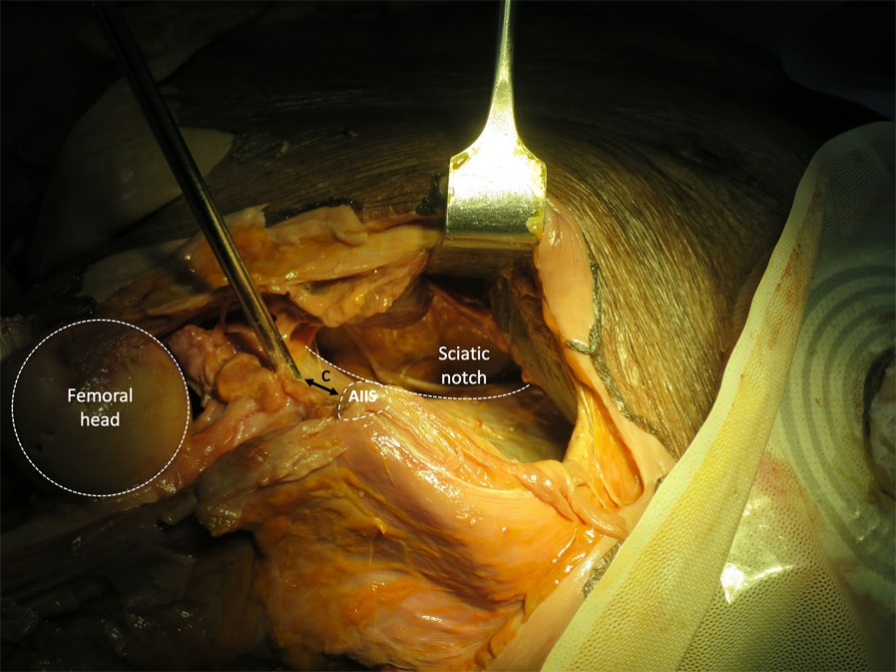
The greater sciatic notch and internal cortex of the ilium are exposed to visualize pin penetration on the left hemipelvis, and the distance (*c*) from the pin to the anterior inferior iliac spine (AIIS)

**Fig. 8:**
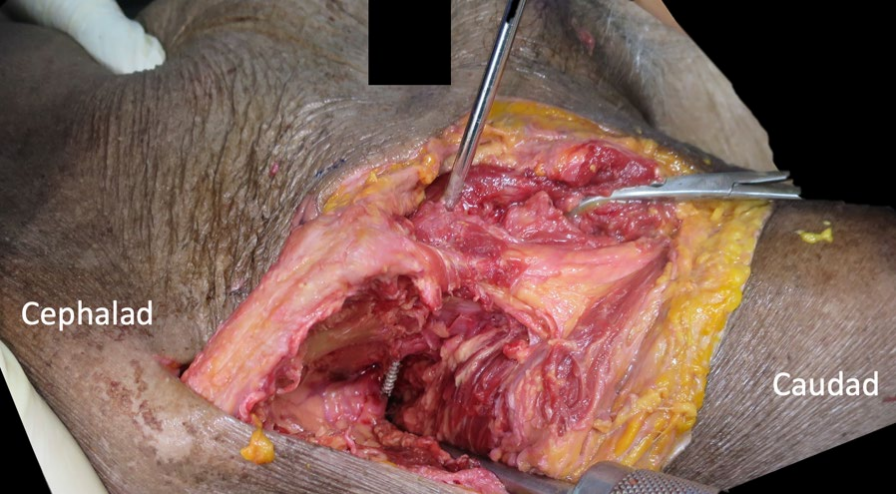
Pin penetration outside the external cortex of the ilium on the right hemipelvis

Open-technique pin placement was compared to standard fluoroscopic guidance in 16 hemipelves (8 fresh-frozen cadavers). The mean age of the donors was 79 years (range 63–89 years, 4 females and 4 males) at the time of death. The open technique was first performed on one side in each cadaver after simple randomization (left and right sequentially); this was followed by fluoroscopic guidance on the other side to prevent bias from visualization of the fluoroscopically guided pin placement. In the open technique group, a 3–4 cm oblique bikini skin incision centered approximately 4 cm distal and 2 cm medial to the anterior superior iliac spine (ASIS) was made (Fig. 1). Initial longitudinal splitting of the fascia over the tensor fasciae latae with artery forceps was followed by digital blunt dissection until the anterior inferior iliac spine (AIIS) could be palpated. A self-tapping Schanz screw (pin) 5 mm in diameter and 200 mm in length (Synthes®; Oberdorf, Solothurn, Switzerland) was inserted in the area just above the AIIS and aimed with a medial inclination of 20° and a cephalad inclination of 10–20° after a 2 cm pilot drill hole had been made using a three-part drill sleeve (Fig. 2). On the opposite hemipelvis, a stab incision using fluoroscopically guided pin placement under the obturator outlet view was achieved by positioning the beam 30° cephalad and 20° medially (Fig. 3). The pin was inserted until the desired pin purchase was obtained by a single experienced trauma surgeon. After both hemipelves had been pinned, three fluoroscopic views were undertaken on both sides in the obturator outlet, iliac oblique, and obturator inlet views (Fig. 4). The LFCN (Fig. 5) and hip capsule (Fig. 6) were identified via the Smith–Peterson approach. Pin penetration outside the bony corridor was clearly seen after complete dissection of soft tissue up to the greater sciatic notch (Fig. 7), and both the internal and external cortex of the ilium were visualized (Fig. 8) to determine if any pins had exited the bony confines of the ilium. The distances from the pin to the LFCN (Fig. 5) and the anterior inferior iliac spine (AIIS) (Fig. 6) and the distance from the capsule of the hip to the acetabulum edge (Fig. 7) were measured in millimeters with a caliper, and pin inclination angles (Fig. 2C, D) were measured using a goniometer, with two observers agreeing on the measurements by consensus. Success was defined as a pin entry point to AIIS distance of within 2 cm. The time required for pin application was recorded.

Statistical analyses were performed using Stata version 14.2 (Stata Corporation, College Station, TX, USA). Continuous variables were expressed using the mean value ± standard deviation (SD) and the range, and categorical variables were expressed as frequencies (percentages). For continuous variable comparisons, Student’s* t*-test for independent samples was used. For categorical variables, Fisher’s exact test was used. All *p*-values were two-sided, and *p*-values < 0.05 were considered statistically significant.

### Source and funding

There was no external source of funding for this study.

## Results

The LFCN was found to be in a risk zone near the pin (mean distance, 15 mm; range, 0–30 mm). One LFCN may have been injured in the fluoroscopic guidance group. The mean medial inclination of the pin was 19.8° (range, 5–40°) and the mean cephalad inclination was 11.5° (range, 0–20°) in the open technique group. The mean medial inclination of the pin was 30.4° (range, 20–45°) and the mean cephalad inclination was 19.3° (range, 2–35°) in the fluoroscopic guidance group. The mean distance of the pin entry point from the AIIS was 11.1 mm (range, 0–35 mm) in the open technique group, while the mean distance of the pin entry point from the AIIS was 15.1 mm (range, 0–25 mm) in the fluoroscopic guidance group. The mean distance from the insertion of the hip capsule to the acetabulum edge was 12 mm (range, 8–25 mm) (Table 1). No joint penetration was observed in the open technique group, compared to one in the fluoroscopic guidance group. No sciatic notch penetration was found in either group, but pin penetration through the external cortex of the ilium was found in 4 hemipelves in the open technique group.

**Table 1:** Results

Pelvis	Side	Technique	LFCN to pin distance (mm)	AIIS to pin distance (mm)	Hip capsule to acetabulum distance (mm)	Medial pin inclination (°)	Cephalad pin inclination (°)
1	L	Open	20	5	12	5	20
	R	PQ	30	25	10	20	20
2	L	PQ	10	20	10	40	20
	R	Open	30	35	25	10	0
3	L	Open	2	20	13	40	20
	R	PQ	15	15	12	45	20
4	L	PQ	21	13	10	38	35
	R	Open	10	0	12	20	10
5	L	Open	20	0	10	21	10
	R	PQ	NA	20	10	21	10
6	L	PQ	15	15	11	23	22
	R	Open	0	20	16	20	5
7	L	Open	4	0	9	22	12
	R	PQ	20	13	12	31	2
8	L	PQ	20	0	8	25	25
	R	Open	10	9	11	20	15
Mean			15	13	12	25	15

*LFCN* lateral femoral cutaneous nerve of the thigh, *AIIS* anteroinferior iliac spine, *PQ* percutaneous, *NA* not found

No complications occurred in the open technique group (0 from 8; 0%), and two complications occurred in the percutaneous group (2 from 8; 25%). We found that the distance from pin entry to the AIIS was within 2 cm (which is defined as successful pin placement) in 7 of the 8 hemipelves (87.5%) in both groups. The mean time required was 4.4 min (range, 3–7 min) in the open technique group and 13.1 min (range, 10–16 min) in the percutaneous technique group (*p* < 0.001) (Table 2).

**Table 2:** Comparison between the two groups

	Open (*n* = 8)	Percutaneous (*n* = 8)	*p*-value
Mean LFCN to pin distance (mm)	12.0 ± 10.5 (0–30)	18.7 ± 6.3 (10–30)	0.164
Unable to identify LFCN	0 (0%)	1 (12.5%)	1.000
Mean medial pin inclination (°)	19.8 ± 10.2 (5–40)	30.4 ± 9.6 (20–45)	0.050
Mean cephalad pin inclination (°)	11.5 ± 6.9 (0–20)	19.3 ± 9.8 (2–35)	0.089
Mean AIIS to pin distance (mm)	11.1 ± 12.8 (0–35)	15.1 ± 7.4 (0–25)	0.456
Entry point of pin within 2 cm of AIIS	7 (87.5%)	7 (87.5%)	1.000
Mean hip capsule to acetabulum distance (mm)	13.5 ± 5.1 (9–25)	10.4 ± 1.3 (8–12)	0.115
Penetration of pin to hip capsule	0 (0%)	1 (12.5%)	1.000
Penetration of pin to sciatic notch	0 (0%)	0 (0%)	1.000
Time required, mean (mins)	4.4 ± 1.3 (3–7)	13.1 ± 1.9 (10–16)	< 0.001

Data expressed as mean ± SD (range) or frequency (percentage)

## Discussion

Standard techniques for identifying the entry point of a supra-acetabular pin using fluoroscopy have been widely used and reported [5–7], and, more recently, ultrasound-assisted entry was utilized in a cadaver study [13]. Due to the superficial structure of the AIIS, which can be palpated, the most accurate guidance tool we have is digital palpation. There is no need for fluoroscopy in unfamiliar views that may reduce the surgical field (especially during utilization of the obturator oblique view, which interferes with the surgeon’s position when applying the Schanz screw) [7, 8], lead to unnecessary radiation exposure, and require extra time.

LFCN injuries are unavoidable due to the inherent variation in this zone of pin placement under both open and fluoroscopic guidance techniques [11], but a mini-open blunt dissection with soft tissue protection technique can minimize these potential injuries [14]. Hip joint penetration is still a risk if pin placement is below the AIIS [8], which can lead to septic arthritis [9]. According to this cadaver study, the open technique without fluoroscopy may not increase the risk of hip joint or sciatic notch penetration compared to fluoroscopic guidance, since we found that one pin in the fluoroscopic guidance group penetrated the hip joint but we did not find any such occurrence in the open technique group. The hip capsule in our study inserted above the acetabulum edge 12 mm proximal to the joint on average (range, 8–25 mm), whereas Haidukewych et al. found that the hip capsule inserted up to 16 mm proximal to the joint on average (range 11–20 mm), and recommended half-pin placement at least 20 mm from the upper edge of the acetabulum [5]. Radiological landmarks related to the AIIS were investigated by Lidder et al. to avoid intra-capsular placement of the pins. They concluded that the pin should be placed in the upper half of the supra-acetabular bone tunnel, which broadly corresponds to the superior half of the AIIS [8]. In this study, we found that hip capsule distance ranged between 8 and 16 mm in 94%, which will not violate the hip capsule if the pin is placed at least 20 mm from the upper edge of the acetabulum, but one hip capsule distance extended up to 25 mm, which may possibly cause pin penetration into the hip joint, so it is reasonable that the pin should be placed in the superior half of the AIIS to avoid intra-capsular placement of the pin. In this study, we found that one pin penetrating into the hip joint was placed in the lower half of the AIIS (Table 1).

We did not encounter sciatic notch penetration, which can cause injury to the superior gluteal neurovascular bundle and lumbosacral trunk [10], because our pin medial inclination angle was not more than 45° in both groups. Pin penetration through the external cortex of the ilium was found in 4 hemipelves in the open technique group, for which the mean pin medial inclination angle was 19.8° (range 5–40°), which was less than in the percutaneous group: 30.4° (range 20–45°). In 2021, Krassnig et al. analyzed computed tomography (CT) scans in trauma patients who were examined with a whole-body CT and had an uninjured pelvic region, and they reported that the angle of insertion of the supra-acetabular pin in the transversal plane (the angle between the Schanz screw and the median sagittal plane) was 21.8° (range 11.7–31.8°) [15]. The pin medial inclination angle of 20° and a cephalad inclination of 10–20° seen in our study cannot be accurately obtained with every pin, just as in clinical practice; these pin angles may vary because of the shape of the AIIS, which causes the pin to tend to slide medially or laterally. Digital palpation of the inner cortex of the ilium to guide the angle may help with pin orientation to prevent inner cortex penetration, but it may not be a reliable reference due to its deep structure, and it cannot represent the proper orientation of the pin or its tendency to exit from the outer cortex of the ilium. Our study indicated that the pin angle itself is most reliable, even for a distorted bony anatomy, due to its large corridor and greater difficulty in exiting the outer cortex earlier, as with an iliac crest pin.

Thus, we recommend that the pin medial inclination should be at least 20° (the lower limit observed for the percutaneous group) to avoid lateral cortex penetration, and should not be more than 45° (the upper limit for both groups) to avoid sciatic notch penetration in the open technique, but these angles may be altered in unstable pelvic fracture patients with a distorted bony anatomy. The pin may extend about 5 cm into the bone, as all the threaded parts of self-tapping Schanz screws anchor inside the cancellous bone and are not long enough to exit from the bony ilium. Krassnig el al. stated that the mean length of the intraosseous part of the Schanz screw in supra-acetabular placement was 80.4 mm, but it was statistically significantly shorter in females, with an average of 74.1 mm (it was 82.7 mm in males) [15].

We do not recommend using an open technique in unstable pelvic fracture patients whose posterior ring stability must be restored and for whom intra-operative fluoroscopy is available, because the accuracy involved in inserting the longest pin toward the posterior inferior iliac spine is unpredictable. However, in cases where only anterior pelvic stabilization is needed, or where fluoroscopy cannot be utilized (such as when a radiolucent operating table is not available, as in a life-saving procedure for an unstable pelvic injury in the emergency room), this open technique takes only one-third of the time required for the percutaneous technique, and is a better way to get adequate stability due to its larger bony corridor than when the pin is placed in the iliac crest. We advocate using this useful technique, as it makes it easier to get adequate stability and, similar to when the pin is placed in the iliac crest, there is no need for fluoroscopic guidance.

There are several limitations of this study. Firstly, pin placement was performed by an experienced trauma surgeon. Secondly, elderly cadaver anatomy cannot be representative of young adult anatomy because of the distorted bony anatomy in pelvic ring injuries. Thirdly, the number of cadavers used in our study is low, but the results are in line with those previously described in the literature [5, 8]. However, all structures at risk should be recognized prior to using this technique, including the femoral artery, which can be palpated medially to the entry point in a patient with a pelvic ring injury.

Additional radiographic studies are necessary to define the proper pin angle to facilitate safe placement of the pin without fluoroscopy in unstable pelvic fracture patients.

## Conclusions

This study shows that a freehand technique performed by experienced trauma surgeons might be as satisfactory as controlled pin insertion under image intensification for selecting the proper entry point and stabilizing the anterior pelvic ring.

## Data Availability

Please contact author for data requests.

## Abbreviations

LFCN: Lateral femoral cutaneous nerve of the thigh
ASIS: Anterior superior iliac spine
AIIS: Anterior inferior iliac spine
